# Crystal structure of chloro­methyl 2-[2-(2,6-di­chloro­phenyl­amino)­phen­yl]acetate

**DOI:** 10.1107/S2056989025004074

**Published:** 2025-05-13

**Authors:** Tobias Keydel, Siva S. M. Bandaru, Lukas Schulig, Andreas Link, Carola Schulzke

**Affiliations:** a Institute of Pharmacy, University of Greifswald, Friedrich-Ludwig-Jahn-Strasse 17, 17489 Greifswald, Germany; b Institute of Biochemistry, University of Greifswald, Felix-Hausdorff-Strasse 4, 17489 Greifswald, Germany; National Taras Shevchenko University of Kyiv, Ukraine

**Keywords:** crystal structure, diclofenac derivative, hydrogen bonding, halogen bonds, halogen-π-inter­actions

## Abstract

The title compound, C_15_H_15_Cl_3_NO_2_, was synthesized from diclofenac and chloro­methyl chloro­sulfate under phase-transfer conditions, and crystallizes in the monoclinic space group *P*2_1_/*c*. As a result of steric strain, the two adjacent aromatic six-membered rings cannot be co-planar, while the terminal ring on one side of the mol­ecule and the methyl acetate moiety atoms on the other reside roughly in the same plane. The angle between the planes of the two aromatic rings is rather wide at 64.27 (8)°. The crystal is tightly packed and consolidated by a large number and notable range of inter­molecular contacts, including relatively strong classical hydrogen bonds but also halogen bonds and even short contacts between chlorine atoms and π-bonds. The inter­molecular inter­actions were further analysed using DFT methods, the results of which are discussed in comparison to the experimental X-ray data.

## Abstract 2

The previously unknown mol­ecular SCXRD structure and crystal-packing pattern of a diclofenac derivative were assessed in detail including intra- and inter­molecular inter­actions such as hydrogen bonds and halogen bonds. The validity of these inter­actions was further evaluated computationally using QM calculations.

## 1. Chemical context

Chloro­methyl esters and their chemical relatives bromo­methyl and iodo­methyl esters are of great synthetic inter­est, as they easily react with carb­oxy­lic acids in the presence of a base to form acyl­als (Keydel & Link, 2024 ▸), which serve as prodrug motifs both for drugs already on the market, *e.g*. sultamicillin (Betrosian & Douzinas, 2009 ▸), pivmecillinam (Burchette *et al.*, 2024 ▸) and clevidipine (Nordlander *et al.*, 2004 ▸), and for experimental compounds of different potential indications (Gao *et al.*, 2022 ▸; Dalaijargal *et al.*, 2022 ▸; Zheng *et al.*, 2022 ▸). During our ongoing investigations into the optimization of the synthesis of acyl­als, we used the chloro­methyl ester of the well-known and widely used NSAID (non-steroidal anti-inflammatory drug) diclofenac as one model substance. It was synthesized from diclofenac in a transesterification reaction with chloro­methyl chloro­sulfate under phase-transfer conditions according to known methods (Binderup & Hansen, 1984 ▸; Harada *et al.*, 1994 ▸).
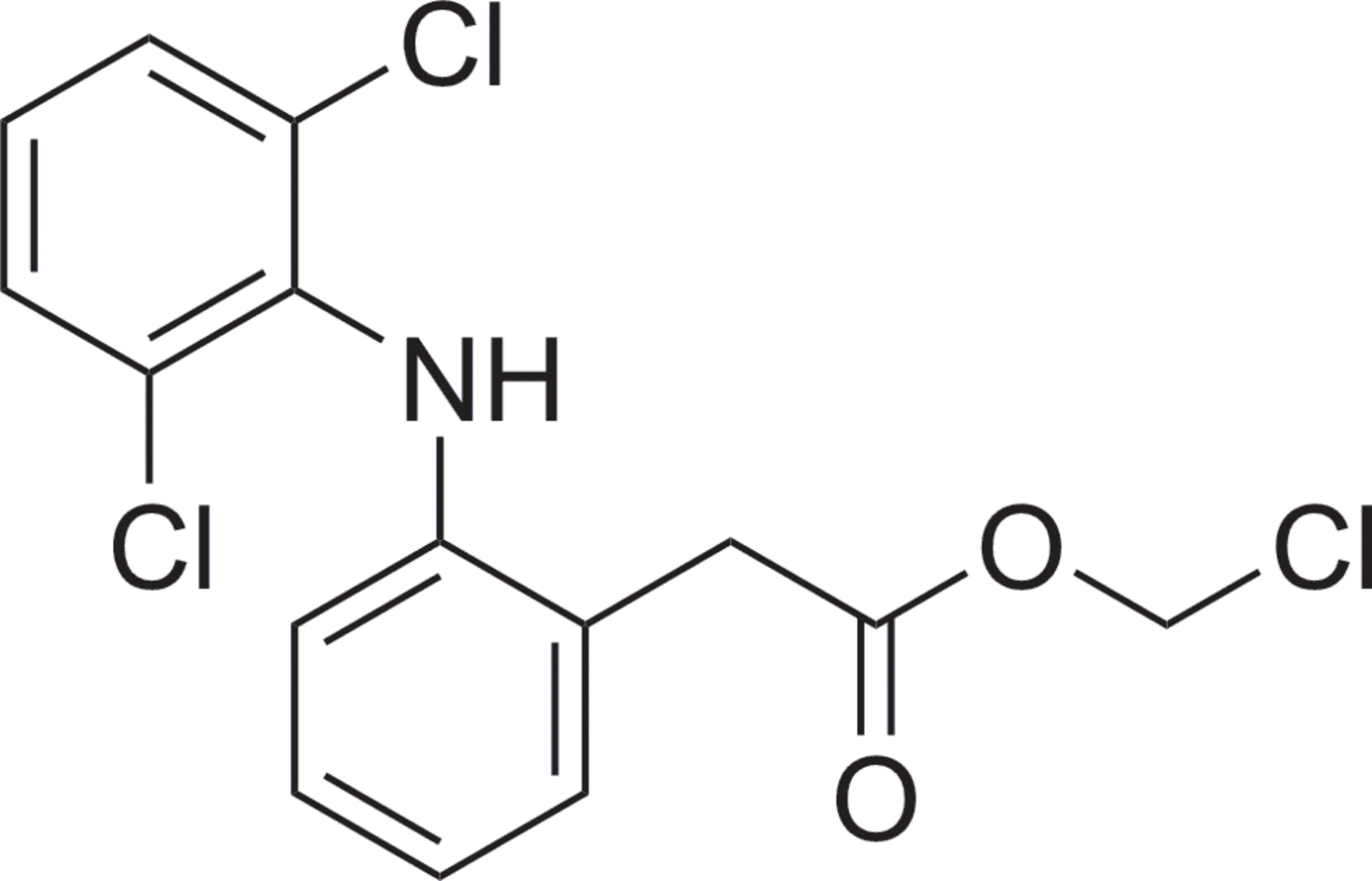


Preparations of the title compound by very similar but not identical procedures were reported before (Dugar *et al.*, 2012 ▸; Xu *et al.*, 2015 ▸), while no characterization data other than low-resolution mass data (*m*/*z* = 344) were reported. The magnificent crystals obtained were characterized and the previously unknown crystal structure is reported herein.

## 2. Structural commentary

**Figure 1:**
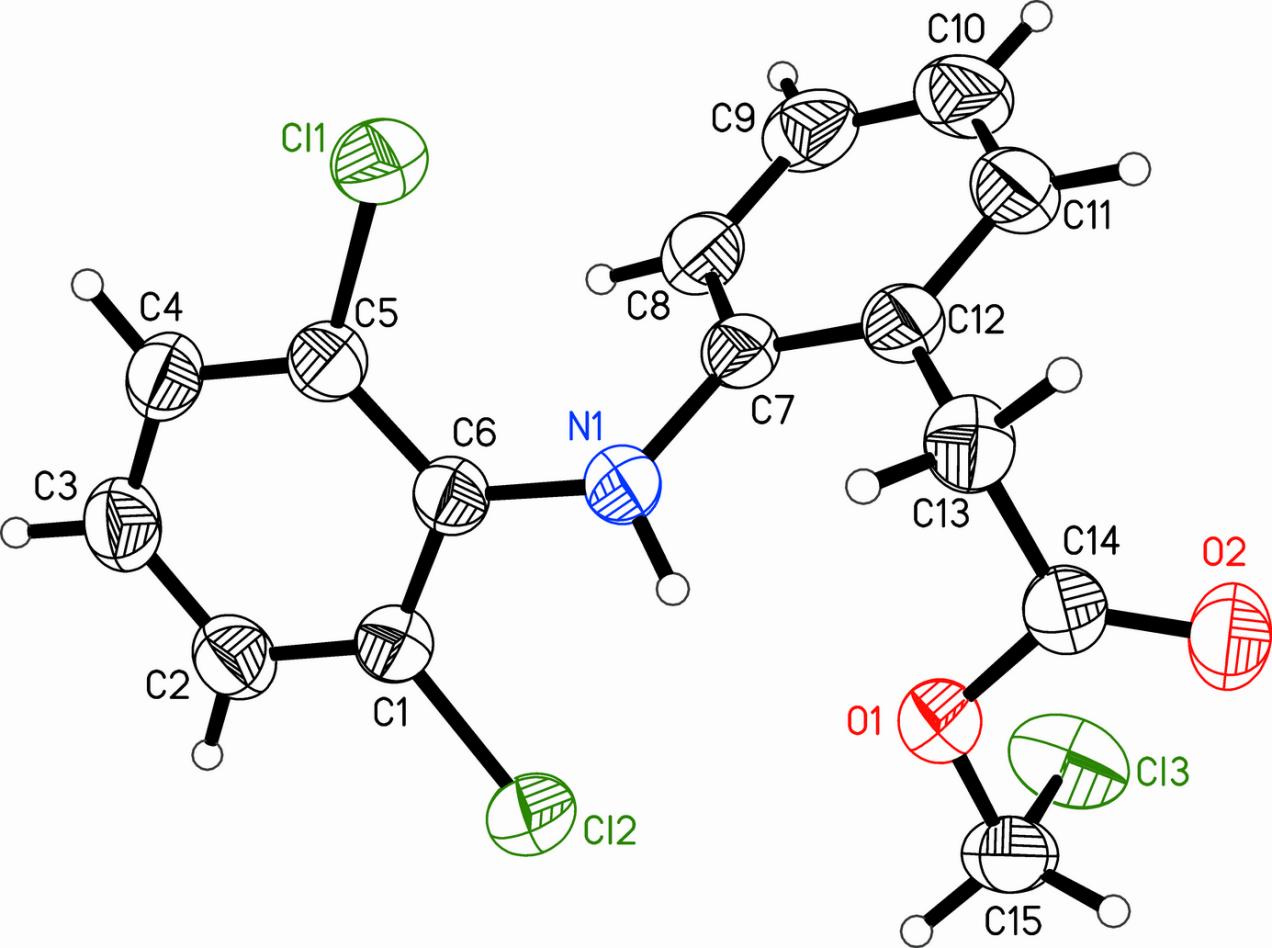
The mol­ecular structure of the title compound chloro­methyl 2-[2-(2,6-di­chloro­phenyl­amino)­phen­yl]acetate. Ellipsoids of the anisotropic atoms are shown at the 50% probability level while the hydrogen atoms have arbitrary radii.

**Figure 2:**
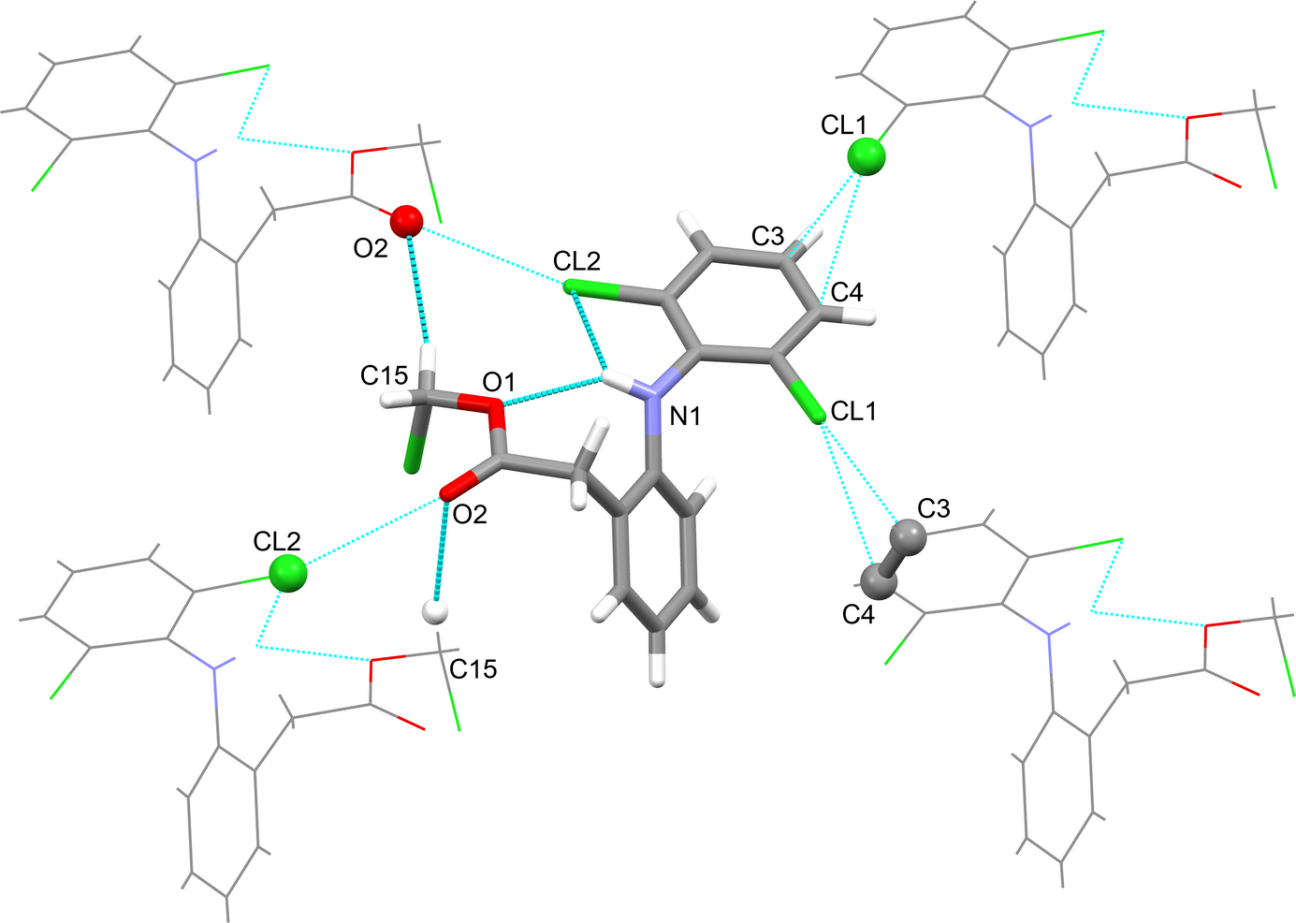
The intra- and inter­molecular inter­actions that support or define the mol­ecule’s geometry and the three-dimensional packing in the crystal. Hydrogen bonds are shown as thick blue lines, halogen bonds and halogen–π inter­actions are shown as thin blue lines. Atoms of the asymmetric unit that are engaged in contacts are labelled.

The mol­ecular structure of the title compound is shown in Fig. 1 ▸. All atoms are in general positions and are all refined in the asymmetric unit. The metrical parameters of bond lengths and angles are inconspicuous as they all fall into the commonly reported ranges. The two aromatic rings are not co-planar but arranged with an angle between the planes (through all six atoms of each ring) of 64.27 (8)°, which is notably wide. This is also evident in the torsion angles involving N1 and C13 as second or third atom (Table 1 ▸). An in-plane situation is hindered by the two chlorine *ortho* substituents of the aniline moiety, while the actually observed torsion between the rings and between the central ring and the ester group is stabilized by two intra­molecular hydrogen bonds with N1—H1*N* as donor to the two acceptors, which are the methyl­ester oxygen atom O1 and the chlorine substituent Cl2 (Table 2 ▸). Fig. 2 ▸ shows all contacts of the asymmetric unit, including the intra­molecular hydrogen bonds. The non-hydrogen atoms of the methyl­acetate chain and the entire aniline moiety lie roughly in the same plane with an angle between the aniline’s six-membered ring and the C13/C14/O1/O2 plane of only 9.78 (8)°. The central phenyl ring (C7–C12) and the terminal chlorine Cl3 point away from the common aniline/acetate plane into the same direction. The nitro­gen atom N1 is slightly pyramidalized with a sum of all three angles of 349.6 ° (see Table 1 ▸ for the angles), which is significantly lower than for a planar nitro­gen with an angle sum of 360°. Again, this geometry is likely supported by the intra­molecular hydrogen bonds (Table 2 ▸).

## 3. Supra­molecular features

The mol­ecules of the title compound in the crystal are engaged in three notable inter­molecular inter­actions (Fig. 2 ▸). These are, firstly, a non-classical hydrogen bond between one methyl­ene hydrogen of the chloro­methyl acetate moiety and the carbonyl oxygen atom of the same moiety of an adjacent mol­ecule (Table 2 ▸; C15—H15*A*⋯O2; symmetry operator: −*x* + 1, *y* − 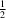, −*z* + 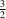). This inter­action is repeated one-directionally so that it protrudes throughout the crystal. It can be classified as a *C*(5) hydrogen-bonding motif (Bernstein *et al.*, 1995 ▸). Secondly, there is a short contact between the terminal chlorine and the carbonyl oxygen atom of a neighbouring mol­ecule, which has a C15—Cl3⋯O1 angle that is sufficiently close to linear at 169.06° (Table 3 ▸; symmetry operator: *x*, 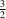 − *y*, −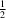 + *z*). This contact may be considered a halogen bond in which the chlorine’s σ-hole opposite to its pivot carbon atom C15 inter­acts with the electron density of a free electron pair of the oxygen (Metrangolo *et al.*, 2008 ▸). Thirdly, there are strikingly close contacts between the aniline substituent Cl1 and the aniline ring carbon atoms C3 and, in particular, C4 of an adjacent mol­ecule (symmetry operator: −*x*, *y* + 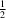, −*z* + 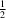). The Cl—C distances and C5—Cl1⋯C3/4 angles are 3.38 Å/177.53° and 3.34 Å/154.79°, respectively. Inter­actions between iodine and aromatic systems were identified spectroscopically as early as 1949 (Benesi & Hildebrand, 1949 ▸). What is observed in the crystal structure of the title compound may be characterized as semi-localized halogen–π bond (Schollmeyer *et al.*, 2008 ▸).

**Figure 3:**
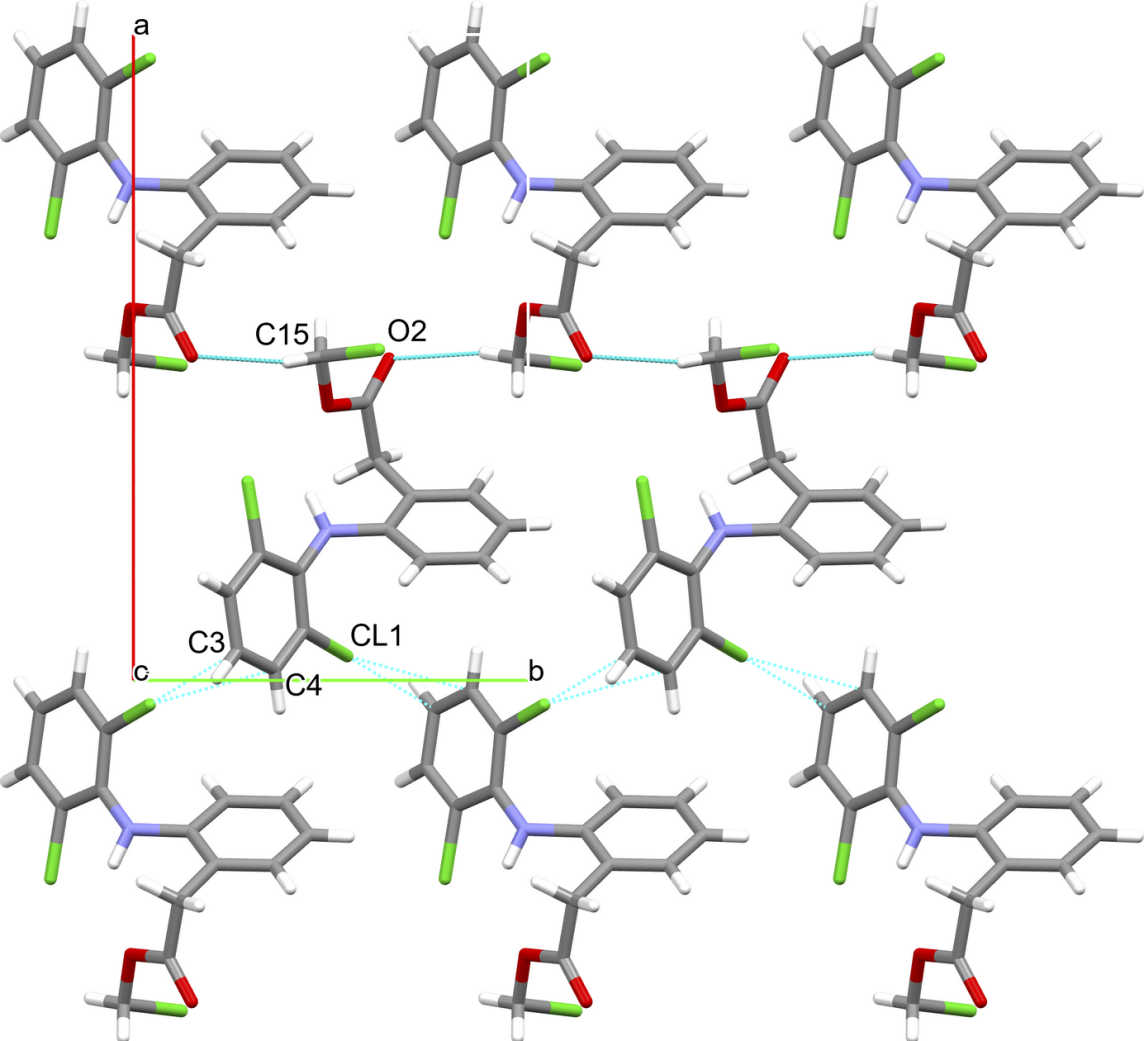
The *C*(5) hydrogen-bonding motif involving the methyl group and the carbonyl oxygen of the methyl­acetate moiety (top) and the halogen–π inter­action (bottom). Both inter­molecular inter­actions protrude parallel to the crystallographic *b*-axis. Labelled atoms belong to the respective asymmetric unit. Hydrogen bonds are shown as thick blue lines, halogen–π inter­actions as thin blue lines. Only atoms of the asymmetric unit are labelled.

**Figure 4:**
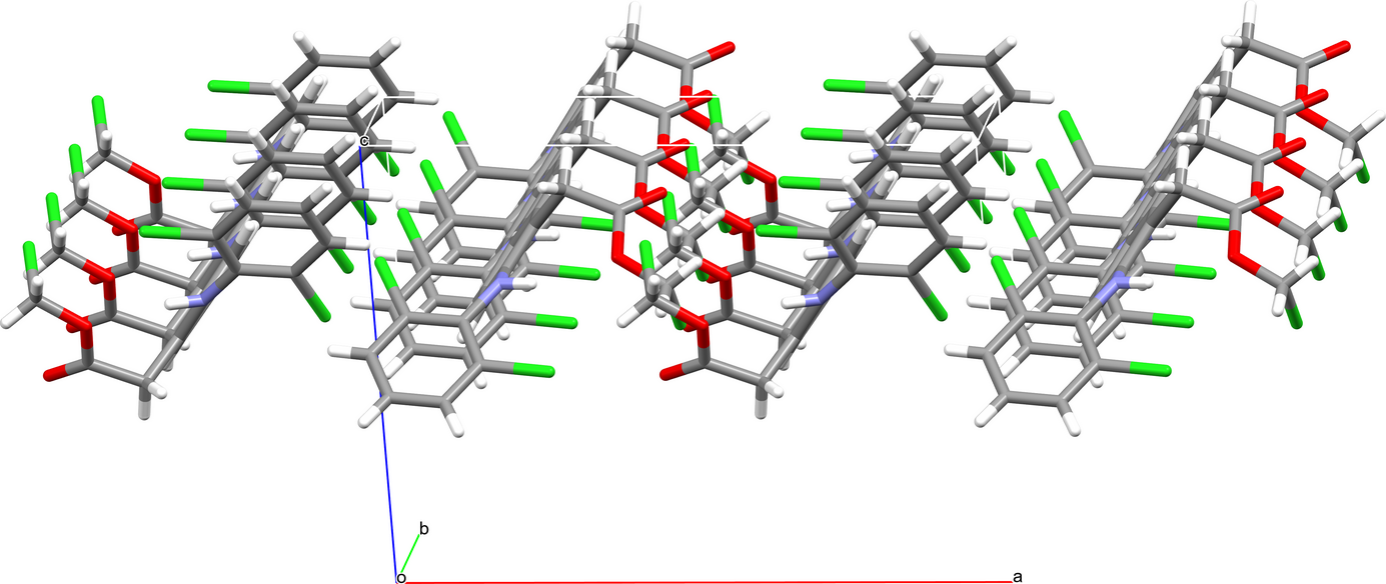
The layer structure formed with a combination of hydrogen bonding and halogen–π inter­action contacts (shown in Fig. 3 ▸) in the crystallographic *ab* plane with a thickness of approximately 0.5 times *c*.

**Figure 5:**
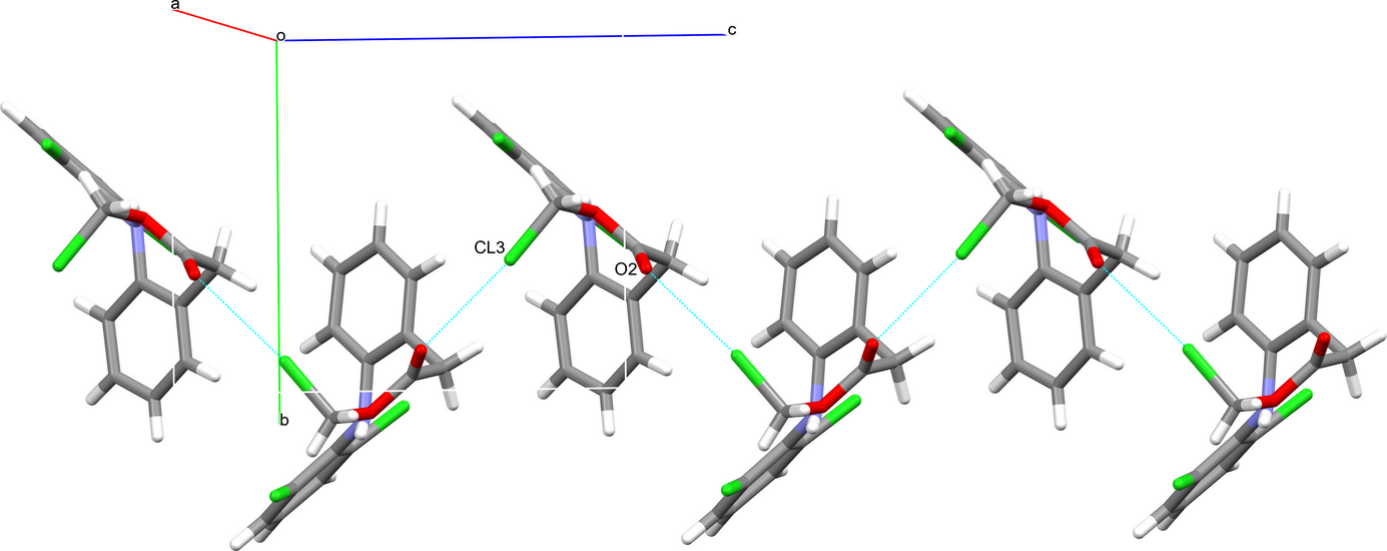
The halogen bond between the terminal chlorine Cl3 and the carbonyl oxygen atom, which protrudes parallel to the crystallographic *c*-axis (contacts shown in blue). This inter­action thereby connects the layer structure shown in Fig. 4 ▸ to the layers above and below, forming a three-dimensional network of contacts that stabilize or define the packing pattern. Only atoms of the asymmetric unit are labelled.

The inter­molecular hydrogen bond and the halogen–π contact both form infinite zigzag chains that protrude in the direction of the crystallographic *b* axis (Fig. 3 ▸, top). The hydrogen bond connects mol­ecules that point alternately up and down parallel to the crystallographic *a* axis. The same is true for the halogen–π contact (Fig. 3 ▸, bottom). Together they thereby form a layer structure in the *ab* plane with an overall thickness of half of the crystallographic *c* axis (Fig. 4 ▸). The inter­actions between the layers, which are stacked in the *c*-axis direction, comprise the halogen bonds between the terminal chlorine Cl3 and the carbonyl oxygen atom of the ester O3, which also protrude in a zigzag pattern (Fig. 5 ▸).

**Figure 6:**
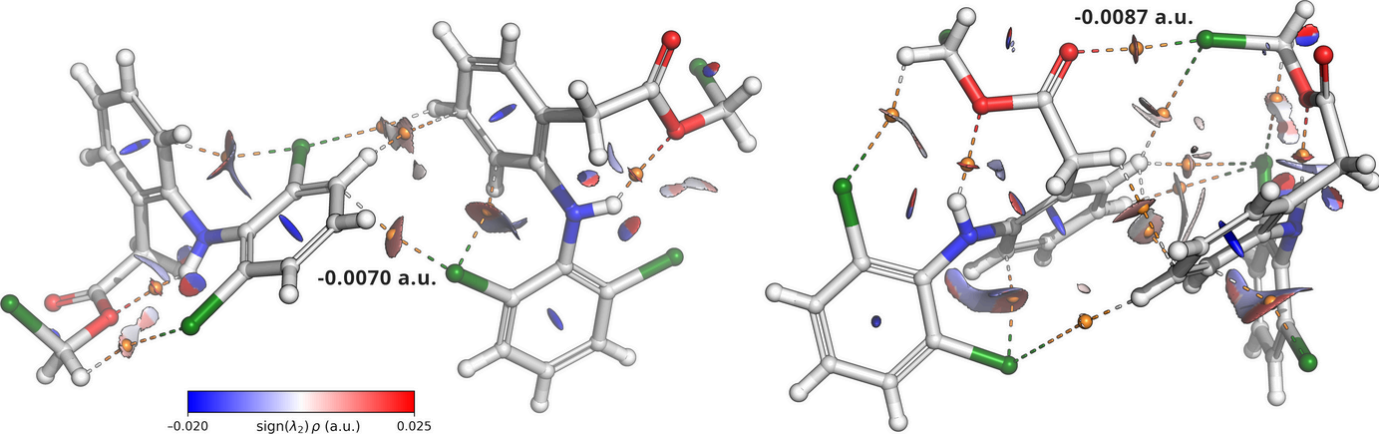
Non-covalent inter­action (NCI) isosurface plot (isovalue = 0.4) of dimers based on the reduced density gradient (RDG) computed from the electron density at the B3LYP-D4/def2-TZVPP level (Schrödinger, 2025 ▸). The orange spheres highlight the bond critical points (BCP) of the identified non-covalent inter­actions.

To support the crystallographically identified inter­molecular contacts, quantum chemical calculations were performed at the B3LYP-D4/def2-TZVPP level of theory using non-covalent inter­action (NCI) analysis based on the reduced density gradient (Fig. 6 ▸). The halogen bond between the terminal chlorine atom Cl3 and the carbonyl oxygen O1 shows a moderately attractive inter­action [sin (λ_2_)ρ = −0.0087 a.u.], consistent with a σ-hole-driven halogen bond. Similarly, the inter­action between Cl1 and the adjacent ring atoms C3 and C4, inter­preted as a halogen–π contact, exhibits a weak, dispersion-driven attraction [sin (λ_2_)ρ = −0.0070 a.u.]. These results align with the crystallographic findings and support the role of these non-covalent inter­actions in stabilizing the supra­molecular assembly.

In addition to the stronger and more apparent non-covalent inter­actions observed there are or may be three weaker contacts present (Table 3 ▸). The first potential inter­action involves C15 and Cl3 and could be inter­preted as a so-called tetrel bond into a π-hole on carbon (Varadwaj *et al.*, 2023 ▸). This runs jointly with the hydrogen bond between O2 and C15 along the crystallographic *b* axis. The second one comprises a short distance between two carbonyl moieties (C14, O2) of adjacent mol­ecules, which are aligned in an inverted parallel arrangement. This may be regarded as a dispersion effect of the two π-systems, since the geometry of the four atoms excludes a tetrel bond-type inter­action. Considering O2 is also involved in relatively stronger hydrogen bonding and a halogen bond, the carbonyl arrangement is likely an effect of the packing rather than that it defines it. The third potential inter­action would be a non-classical hydrogen bond C2—H2⋯N1 (Table 2 ▸) protruding along the crystallographic *c* axis. Considering the distances between the respective C, H and N atoms and the fact that the adjacent halogen – contact also runs along the *c*-axis direction in the exact same zigzag manner, it is more likely that the former arrangement is a consequence of the more prominent and stronger latter one. We do not consider these three weaker contacts to be of particular importance for the overall packing.

Despite the presence of two aromatic six-membered rings, no aromatic π–π inter­actions are observed with metrical parameters (distance, slippage *etc*.) that would suggest any relevance for the packing pattern.

## 4. Database survey

The CSD (Groom *et al.*, 2016 ▸) was searched using the ConQuest programme version 2024.2.0 (Bruno *et al.*, 2002 ▸), as part of the CSD software suite. A search for the entire backbone (aniline, phenyl ring, and acetate moiety) resulted in 186 hits. Limiting the search by the introduction of the two chlorine substituents on the aniline ring, which are characteristic for diclofenac, reduced the hits by only 6 to 180, which is quite notable. To further narrow down the hit list to mol­ecules that would be well comparable to the title compound, the methyl carbon of the ester group was included. This search returned 29 hits of which 7 are repeatedly reported, *i.e.* 22 distinct mol­ecular structures in the database. From these were excluded metal-bearing hits, those with relatively large substituents such as sugars, and those with another not covalently bound component in the crystal, *e.g*., solvent. This resulted in 14 hits and 7 individual structures distinct in the substituents on the methyl­acetate moiety, which were assessed in comparison with the title compound. One general defining structural feature is the torsion of the central phenyl ring relative to the aniline moiety. A change in torsion direction results in the aniline and the acetate moiety swapping places when looking at the central ring as leaning away from the viewer, while the aniline and acetate moieties lie in the horizontal plane. In the title compound, the torsion angle C6—N1—C7—C12 is 166.62 (13)° and the di­chloro­aniline ring appears on the right-hand side of the reclining central phenyl ring. This is also the case for the acet­oxy­acetic acid derivative with a torsion of 178.8° involving the analogous four atoms (BOXMUZ; Jelsch *et al.*, 2020 ▸), for the ethyl­acetate derivative with *ca*. 164.5° analogous torsion in all six reported structures (DUBWAB and DUBWAB01–05; Nugrahani *et al.*, 2019 ▸; Chodkiewicz *et al.*, 2022 ▸), and the methyl­acetate derivative with 165.1° (XEYZIL01; Saleem *et al.*, 2008 ▸). For the latter, a second structure is published in a different crystal system and space group for which the torsion of analogous atoms occurs in opposite direction with −178.3° (XEYZIL; Nawaz *et al.*, 2007 ▸) and the di­chloro­aniline moiety appears to the left-hand side of the viewer looking at the reclining central phenyl ring. That is also the case for a mixed structure of diclofenac together with its ethyl­acetate derivative with both mol­ecules having negative analogous torsions of −161.5 and −173.6° (DUBWEF; Nugrahani *et al.*, 2019 ▸), respectively, the 2-propyl­acetate derivative with −167.0° (MISBEW; Nawaz *et al.*, 2008 ▸), the 4-nitro-aniline and 3,5-di­nitro­phenyl derivative with a torsion of −148.0° (UNUDEN; Tariq *et al.*, 2011 ▸), and another acet­oxy­acetic acid derivative, which is a respective torsional isomer of BOXMUZ with a torsion angle of −172.8° and −173.2° in the two reported structures (VUGCUV and VUGCUV01; Alvarez-Larena *et al.*, 1992 ▸; Goud *et al.*, 2013 ▸). All observed absolute torsion angle values fall into a relatively narrow range of 160–180°, which emphasizes a certain joint geometric rigidity of the two aromatic rings, likely enforced by steric strain from the *ortho*-chlorine substituents and the intra­molecular hydrogen bonding involving the NH group. In the title compound and in XEYZIL it is the substituted oxygen that points toward the nitro­gen atom for an intra­molecular hydrogen bond. In all other cases it is the carbonyl oxygen. This orientation is therefore somehow unusual and as a result, these two are the only examples in which the carbonyl oxygen atom is free to engage in inter­molecular hydrogen bonding, which the substituted oxygen does not in any of the other assessed examples. As a result, the inter­molecular inter­actions of the title compound chloro­methyl 2-[2-(2,6-di­chloro­phenyl­amino)­phen­yl]acetate are particularly rich and three-dimensional, which facilitates a unique packing pattern amongst these derivatives.

## 5. Synthesis and crystallization

To a solution of diclofenac (1.481 g, 5.0 mmol, 1.00 equiv.), sodium hydrogen carbonate (1.260 g, 15.0 mmol, 3.00 equiv.) and tetra­butyl­ammonium hydrogen sulfate (162 mg, 0.5 mmol, 0.10 equiv.) in water (50 mL) and di­chloro­methane (10 mL), a solution of chloro­methyl chloro­sulfate (0.759 mL, 7.5 mmol, 1.50 equiv.) in di­chloro­methane (40 mL) was added dropwise at 273 K over a period of 1 h. The ice bath was removed, and the reaction was allowed to warm to room temperature and left to stir for 16 h. After completion of the reaction (monitored by TLC analysis of the organic phase), the organic phase was separated and washed with saturated sodium hydrogen carbonate solution (3×) and brine. After drying over MgSO_4_, the solvent was removed under reduced pressure and the residue was purified by dry column vacuum chromatography (0–20% ethyl acetate in hexa­ne) yielding a white solid. Crystallization was accomplished from methanol yielding the title compound as colourless rhomboid crystals (yield 1.325 g, 3.845 mmol, 77%): *R_f_* = 0.24 (hexa­ne); m.p. 359 K; ^1^H-NMR (400 MHz, DMSO-*d*_6_) δ 7.79–7.72 (*m*, 2H), 7.62 (*dd*, *J* = 7.5 Hz, 1H), 7.39 (*d*, *J* = 7.4 Hz, 1H), 7.24–7.18 (*m*, 1H), 7.09 (*td*, *J* = 7.5 Hz, 1H), 6.39 (*d*, *J* = 7.3 Hz, 1H), 3.89 (*s*, 2H).; ^13^C NMR (101 MHz, DMSO-*d*6) δ 169.70, 142.98, 137.00, 131.28, 131.05, 129.12, 127.99, 126.23, 121.88, 120.41, 115.60, 69.74, 36.33; ESI-HRMS: *m*/*z* calculated for [C_15_H_12_^35^Cl_3_NO_2_ + H]^+^ 344.0006, found: 344.0001. Purity: 99.4% (254 nm). General experimental information such as the employed instruments as well as the NMR spectra can be found in the supporting information.

## 6. Refinement

Crystal data, data collection and structure refinement details are summarized in Table 4 ▸. All hydrogen atoms were located and refined freely.

## Supplementary Material

Crystal structure: contains datablock(s) I. DOI: 10.1107/S2056989025004074/nu2009sup1.cif

Structure factors: contains datablock(s) I. DOI: 10.1107/S2056989025004074/nu2009Isup2.hkl

General experimental information such as the employed instruments as well as the NMR spectra can be found in the Supplementary Information file. DOI: 10.1107/S2056989025004074/nu2009sup3.pdf

Supporting information file. DOI: 10.1107/S2056989025004074/nu2009Isup4.cml

CCDC reference: 2449037

Additional supporting information:  crystallographic information; 3D view; checkCIF report

## Figures and Tables

**Table 1:** Selected bond and torsion angles (°)

C7—N1—H1*N*	113.6 (12)	C2—C1—Cl2	118.39 (12)
C2—C1—C6	122.66 (13)		
			
C7—N1—C6—C1	124.89 (15)	C11—C12—C13—C14	−90.93 (16)
C7—N1—C6—C5	−59.6 (2)	C7—C12—C13—C14	87.17 (16)
C6—N1—C7—C8	−13.4 (2)	C12—C13—C14—O2	104.07 (17)
C6—N1—C7—C12	166.62 (13)	C12—C13—C14—O1	−73.22 (15)

**Table 2:** Hydrogen-bond geometry (Å, °)

*D*—H⋯*A*	*D*—H	H⋯*A*	*D*⋯*A*	*D*—H⋯*A*
N1—H1*N*⋯O1	0.87 (2)	2.217 (19)	2.9568 (15)	142.2 (16)
N1—H1*N*⋯Cl2	0.87 (2)	2.536 (19)	2.9890 (13)	113.1 (15)
C15—H15*A*⋯O2^i^	0.95 (2)	2.59 (2)	3.521 (2)	168.2 (15)
C2—H2⋯N1^ii^	0.96 (2)	2.93 (2)	3.886 (2)	173.6 (16)

Symmetry codes: (i) 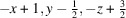; (ii) 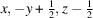.

**Table 3:** Non-covalent inter­molecular contacts (Å, °)

Contact	Operator	Distance	Geometry	Angle
Cl3⋯O2	*x*, −*y* + 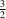 , *z* − 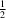	3.066 (12)	C15—Cl3⋯O2	169.1 (07)
Cl1⋯C3	−*x*, *y* + 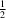 , −*z* + 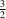	3.385 (18)	C5—Cl1⋯C3	177.6 (06)
Cl1⋯C4	−*x*, *y* + 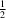 , −*z* + 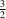	3.340 (16)	C5—Cl1⋯C4	154.8 (06)
C14⋯O2	−*x* + 1, −*y* + 1, −*z* + 2	3.044 (18)	O2—C14⋯O2	82.3 (09)
Cl3⋯C15	−*x* + 1, *y* + 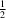 , −*z* + 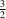	3.577 (17)	C15—Cl3⋯C15	115.8 (03)

**Table 4:** Experimental details

Crystal data	
Chemical formula	C_15_H_12_Cl_3_NO_2_
*M*_r_	344.61
Crystal system, space group	Monoclinic, *P*2_1_/*c*
Temperature (K)	299
*a*, *b*, *c* (Å)	15.1487 (1), 9.2389 (1), 10.8661 (1)
β (°)	94.242 (1)
*V* (Å^3^)	1516.62 (2)
*Z*	4
Radiation type	Cu *K*α
μ (mm^−1^)	5.50
Crystal size (mm)	0.18 × 0.13 × 0.10

Data collection	
Diffractometer	XtaLAB Synergy, Single source at home/near, HyPix
Absorption correction	Numerical (face indexed; *CrysAlis PRO*, Rigaku OD, 2024 ▸)
*T*_min_, *T*_max_	0.507, 0.856
No. of measured, independent and observed [*I* > 2σ(*I*)] reflections	54733, 3301, 3145
*R*_int_	0.031
(sin θ/λ)_max_ (Å^−1^)	0.639

Refinement	
*R*[*F*^2^ > 2σ(*F*^2^)], *wR*(*F*^2^), *S*	0.031, 0.081, 1.07
No. of reflections	3301
No. of parameters	238
H-atom treatment	All H-atom parameters refined
Δρ_max_, Δρ_min_ (e Å^−3^)	0.34, −0.46

Computer programs: *CrysAlis PRO* (Rigaku OD, 2024 ▸), *SHELXT2018/3* (Sheldrick, 2015*a* ▸), *SHELXL2019/2* (Sheldrick, 2015*b* ▸), *XP* in *SHELXTL* (Sheldrick, 2008 ▸), *Mercury* (Macrae *et al.*, 2020 ▸), *CIFTAB* (Sheldrick, 2015*b* ▸) and *publCIF* (Westrip, 2010 ▸).

## supplementary crystallographic information

### Chloromethyl 2-[2-(2,6-dichlorophenylamino)phenyl]acetate Crystal data

**Table:**

C_15_H_12_Cl_3_NO_2_	*F*(000) = 704
*M**_r_* = 344.61	*D*_x_ = 1.509 Mg m^−^^3^
Monoclinic, *P*2_1_/*c*	Cu *K*α radiation, λ = 1.54184 Å
*a* = 15.1487 (1) Å	Cell parameters from 37256 reflections
*b* = 9.2389 (1) Å	θ = 5.1–79.6°
*c* = 10.8661 (1) Å	µ = 5.50 mm^−^^1^
β = 94.242 (1)°	*T* = 299 K
*V* = 1516.62 (2) Å^3^	Block, colourless
*Z* = 4	0.18 × 0.13 × 0.10 mm

### Chloromethyl 2-[2-(2,6-dichlorophenylamino)phenyl]acetate Data collection

**Table:**

XtaLAB Synergy, Single source at home/near, HyPix diffractometer	3301 independent reflections
Radiation source: micro-focus sealed X-ray tube	3145 reflections with *I* > 2σ(*I*)
Detector resolution: 10.0000 pixels mm^-1^	*R*_int_ = 0.031
ω scans	θ_max_ = 80.1°, θ_min_ = 5.6°
Absorption correction: numerical (face indexed; CrysAlisPro, Rigaku OD, 2024)	*h* = −19→19
*T*_min_ = 0.507, *T*_max_ = 0.856	*k* = −11→11
54733 measured reflections	*l* = −12→13

### Chloromethyl 2-[2-(2,6-dichlorophenylamino)phenyl]acetate Refinement

**Table:**

Refinement on *F*^2^	Primary atom site location: dual
Least-squares matrix: full	Secondary atom site location: difference Fourier map
*R*[*F*^2^ > 2σ(*F*^2^)] = 0.031	Hydrogen site location: difference Fourier map
*wR*(*F*^2^) = 0.081	All H-atom parameters refined
*S* = 1.07	*w* = 1/[σ^2^(*F*_o_^2^) + (0.0387*P*)^2^ + 0.4573*P*] where *P* = (*F*_o_^2^ + 2*F*_c_^2^)/3
3301 reflections	(Δ/σ)_max_ = 0.001
238 parameters	Δρ_max_ = 0.34 e Å^−^^3^
0 restraints	Δρ_min_ = −0.46 e Å^−^^3^

### Chloromethyl 2-[2-(2,6-dichlorophenylamino)phenyl]acetate Special details

**Table:**

Geometry. All esds (except the esd in the dihedral angle between two l.s. planes) are estimated using the full covariance matrix. The cell esds are taken into account individually in the estimation of esds in distances, angles and torsion angles; correlations between esds in cell parameters are only used when they are defined by crystal symmetry. An approximate (isotropic) treatment of cell esds is used for estimating esds involving l.s. planes.

### Chloromethyl 2-[2-(2,6-dichlorophenylamino)phenyl]acetate Fractional atomic coordinates and isotropic or equivalent isotropic displacement parameters (Å^2^)

**Table:**

	*x*	*y*	*z*	*U*_iso_*/*U*_eq_	
Cl1	0.03768 (2)	0.54114 (4)	0.78340 (4)	0.05292 (12)	
Cl2	0.30724 (3)	0.28951 (5)	0.56128 (5)	0.06261 (13)	
Cl3	0.51214 (4)	0.62138 (5)	0.63287 (4)	0.06918 (15)	
O1	0.42456 (7)	0.49277 (11)	0.80019 (10)	0.0451 (2)	
O2	0.49799 (7)	0.64736 (13)	0.93006 (11)	0.0539 (3)	
N1	0.23090 (8)	0.49080 (13)	0.74080 (12)	0.0430 (3)	
C1	0.19495 (9)	0.31935 (15)	0.57362 (14)	0.0424 (3)	
C2	0.13490 (11)	0.24256 (17)	0.49680 (15)	0.0491 (3)	
C3	0.04534 (11)	0.26343 (18)	0.50614 (16)	0.0520 (4)	
C4	0.01648 (10)	0.35706 (17)	0.59320 (16)	0.0479 (3)	
C5	0.07738 (9)	0.43110 (15)	0.67077 (13)	0.0408 (3)	
C6	0.16882 (9)	0.41802 (14)	0.66173 (13)	0.0385 (3)	
C7	0.23400 (8)	0.64393 (14)	0.75110 (13)	0.0387 (3)	
C8	0.18753 (10)	0.73305 (17)	0.66555 (16)	0.0468 (3)	
C9	0.18976 (11)	0.88217 (18)	0.67993 (19)	0.0559 (4)	
C10	0.23924 (13)	0.94408 (18)	0.7780 (2)	0.0611 (5)	
C11	0.28691 (11)	0.85629 (17)	0.86162 (17)	0.0529 (4)	
C12	0.28521 (9)	0.70601 (15)	0.85000 (13)	0.0408 (3)	
C13	0.34086 (10)	0.61469 (18)	0.94222 (14)	0.0451 (3)	
C14	0.43033 (9)	0.58920 (15)	0.89501 (12)	0.0400 (3)	
C15	0.50149 (11)	0.47487 (18)	0.73558 (16)	0.0496 (3)	
H2	0.1567 (13)	0.178 (2)	0.4372 (19)	0.060 (5)*	
H3	0.0032 (14)	0.212 (2)	0.4547 (19)	0.066 (6)*	
H4	−0.0433 (14)	0.370 (2)	0.6010 (18)	0.060 (5)*	
H8	0.1548 (13)	0.687 (2)	0.5951 (19)	0.060 (5)*	
H9	0.1590 (14)	0.939 (2)	0.621 (2)	0.065 (6)*	
H10	0.2419 (15)	1.045 (3)	0.785 (2)	0.081 (7)*	
H11	0.3225 (13)	0.897 (2)	0.9322 (19)	0.060 (5)*	
H13A	0.3497 (12)	0.666 (2)	1.0206 (17)	0.051 (5)*	
H13B	0.3129 (12)	0.522 (2)	0.9566 (16)	0.050 (5)*	
H15A	0.4927 (12)	0.390 (2)	0.6869 (18)	0.054 (5)*	
H15B	0.5529 (14)	0.474 (2)	0.7902 (18)	0.056 (5)*	
H1N	0.2831 (13)	0.450 (2)	0.7451 (17)	0.053 (5)*	

### Chloromethyl 2-[2-(2,6-dichlorophenylamino)phenyl]acetate Atomic displacement parameters (Å^2^)

**Table:**

	*U*^11^	*U*^22^	*U*^33^	*U*^12^	*U*^13^	*U*^23^
Cl1	0.0467 (2)	0.0523 (2)	0.0613 (2)	0.00247 (15)	0.01461 (16)	−0.00421 (16)
Cl2	0.0417 (2)	0.0615 (3)	0.0856 (3)	0.00329 (16)	0.01117 (18)	−0.0195 (2)
Cl3	0.0967 (4)	0.0564 (2)	0.0574 (2)	0.0089 (2)	0.0263 (2)	0.00548 (18)
O1	0.0401 (5)	0.0443 (5)	0.0507 (6)	0.0000 (4)	0.0028 (4)	−0.0075 (4)
O2	0.0447 (6)	0.0593 (7)	0.0565 (6)	−0.0093 (5)	−0.0037 (5)	−0.0064 (5)
N1	0.0365 (6)	0.0359 (6)	0.0552 (7)	0.0023 (5)	−0.0060 (5)	−0.0036 (5)
C1	0.0393 (7)	0.0378 (7)	0.0502 (8)	0.0005 (5)	0.0035 (6)	0.0008 (6)
C2	0.0536 (8)	0.0431 (7)	0.0502 (8)	−0.0010 (6)	0.0009 (6)	−0.0058 (6)
C3	0.0491 (8)	0.0467 (8)	0.0584 (9)	−0.0069 (7)	−0.0087 (7)	−0.0021 (7)
C4	0.0365 (7)	0.0447 (8)	0.0616 (9)	−0.0029 (6)	−0.0017 (6)	0.0051 (7)
C5	0.0390 (7)	0.0358 (6)	0.0476 (7)	0.0003 (5)	0.0037 (5)	0.0037 (5)
C6	0.0372 (6)	0.0333 (6)	0.0447 (7)	−0.0003 (5)	−0.0002 (5)	0.0032 (5)
C7	0.0333 (6)	0.0353 (6)	0.0481 (7)	0.0000 (5)	0.0075 (5)	0.0001 (5)
C8	0.0380 (7)	0.0456 (8)	0.0567 (9)	0.0012 (6)	0.0041 (6)	0.0069 (7)
C9	0.0456 (8)	0.0446 (8)	0.0785 (11)	0.0067 (7)	0.0120 (8)	0.0160 (8)
C10	0.0606 (10)	0.0349 (8)	0.0899 (13)	0.0011 (7)	0.0189 (9)	−0.0002 (8)
C11	0.0522 (8)	0.0423 (8)	0.0654 (10)	−0.0045 (6)	0.0131 (7)	−0.0112 (7)
C12	0.0368 (6)	0.0400 (7)	0.0466 (7)	−0.0012 (5)	0.0098 (5)	−0.0039 (5)
C13	0.0459 (7)	0.0500 (8)	0.0398 (7)	−0.0042 (6)	0.0050 (6)	−0.0016 (6)
C14	0.0433 (7)	0.0374 (6)	0.0386 (6)	−0.0010 (5)	−0.0016 (5)	0.0035 (5)
C15	0.0484 (8)	0.0429 (8)	0.0582 (9)	0.0090 (6)	0.0082 (7)	−0.0009 (7)

### Chloromethyl 2-[2-(2,6-dichlorophenylamino)phenyl]acetate Geometric parameters (Å, º)

**Table:**

Cl1—C5	1.7325 (15)	C5—C6	1.4012 (19)
Cl2—C1	1.7381 (14)	C7—C8	1.393 (2)
Cl3—C15	1.7693 (17)	C7—C12	1.401 (2)
O1—C14	1.3600 (17)	C8—C9	1.387 (2)
O1—C15	1.4141 (18)	C8—H8	0.98 (2)
O2—C14	1.1945 (17)	C9—C10	1.381 (3)
N1—C6	1.3983 (17)	C9—H9	0.92 (2)
N1—C7	1.4197 (17)	C10—C11	1.381 (3)
N1—H1N	0.87 (2)	C10—H10	0.94 (2)
C1—C2	1.384 (2)	C11—C12	1.394 (2)
C1—C6	1.400 (2)	C11—H11	0.98 (2)
C2—C3	1.381 (2)	C12—C13	1.517 (2)
C2—H2	0.96 (2)	C13—C14	1.503 (2)
C3—C4	1.377 (2)	C13—H13A	0.974 (19)
C3—H3	0.95 (2)	C13—H13B	0.974 (19)
C4—C5	1.384 (2)	C15—H15A	0.95 (2)
C4—H4	0.92 (2)	C15—H15B	0.94 (2)
			
C14—O1—C15	116.33 (12)	C10—C9—C8	120.39 (16)
C6—N1—C7	122.99 (12)	C10—C9—H9	120.8 (13)
C6—N1—H1N	113.0 (12)	C8—C9—H9	118.8 (13)
C7—N1—H1N	113.6 (12)	C9—C10—C11	119.47 (15)
C2—C1—C6	122.66 (13)	C9—C10—H10	119.8 (14)
C2—C1—Cl2	118.39 (12)	C11—C10—H10	120.7 (15)
C6—C1—Cl2	118.93 (11)	C10—C11—C12	121.33 (17)
C3—C2—C1	119.30 (15)	C10—C11—H11	121.4 (12)
C3—C2—H2	121.8 (12)	C12—C11—H11	117.3 (12)
C1—C2—H2	118.9 (12)	C11—C12—C7	118.86 (14)
C4—C3—C2	120.11 (15)	C11—C12—C13	119.23 (14)
C4—C3—H3	119.3 (13)	C7—C12—C13	121.88 (13)
C2—C3—H3	120.6 (13)	C14—C13—C12	109.44 (12)
C3—C4—C5	119.86 (14)	C14—C13—H13A	107.7 (11)
C3—C4—H4	120.6 (12)	C12—C13—H13A	110.0 (11)
C5—C4—H4	119.6 (12)	C14—C13—H13B	109.3 (11)
C4—C5—C6	122.19 (14)	C12—C13—H13B	111.6 (11)
C4—C5—Cl1	118.02 (11)	H13A—C13—H13B	108.7 (15)
C6—C5—Cl1	119.79 (11)	O2—C14—O1	122.81 (14)
N1—C6—C1	121.46 (12)	O2—C14—C13	126.51 (14)
N1—C6—C5	122.61 (13)	O1—C14—C13	110.62 (12)
C1—C6—C5	115.79 (12)	O1—C15—Cl3	109.92 (10)
C8—C7—C12	119.59 (13)	O1—C15—H15A	106.6 (12)
C8—C7—N1	121.61 (13)	Cl3—C15—H15A	107.2 (12)
C12—C7—N1	118.80 (12)	O1—C15—H15B	111.2 (12)
C9—C8—C7	120.34 (16)	Cl3—C15—H15B	107.3 (12)
C9—C8—H8	121.7 (12)	H15A—C15—H15B	114.6 (16)
C7—C8—H8	118.0 (12)		
			
C6—C1—C2—C3	0.2 (2)	C12—C7—C8—C9	−1.9 (2)
Cl2—C1—C2—C3	179.24 (13)	N1—C7—C8—C9	178.14 (14)
C1—C2—C3—C4	−1.6 (3)	C7—C8—C9—C10	1.1 (2)
C2—C3—C4—C5	0.5 (2)	C8—C9—C10—C11	0.3 (3)
C3—C4—C5—C6	2.1 (2)	C9—C10—C11—C12	−0.8 (3)
C3—C4—C5—Cl1	−177.06 (12)	C10—C11—C12—C7	0.1 (2)
C7—N1—C6—C1	124.89 (15)	C10—C11—C12—C13	178.24 (15)
C7—N1—C6—C5	−59.6 (2)	C8—C7—C12—C11	1.3 (2)
C2—C1—C6—N1	177.97 (14)	N1—C7—C12—C11	−178.74 (13)
Cl2—C1—C6—N1	−1.03 (19)	C8—C7—C12—C13	−176.84 (13)
C2—C1—C6—C5	2.1 (2)	N1—C7—C12—C13	3.2 (2)
Cl2—C1—C6—C5	−176.86 (10)	C11—C12—C13—C14	−90.93 (16)
C4—C5—C6—N1	−179.08 (13)	C7—C12—C13—C14	87.17 (16)
Cl1—C5—C6—N1	0.05 (19)	C15—O1—C14—O2	−6.3 (2)
C4—C5—C6—C1	−3.3 (2)	C15—O1—C14—C13	171.15 (12)
Cl1—C5—C6—C1	175.83 (10)	C12—C13—C14—O2	104.07 (17)
C6—N1—C7—C8	−13.4 (2)	C12—C13—C14—O1	−73.22 (15)
C6—N1—C7—C12	166.62 (13)	C14—O1—C15—Cl3	−77.80 (14)

### Chloromethyl 2-[2-(2,6-dichlorophenylamino)phenyl]acetate Hydrogen-bond geometry (Å, º)

**Table:**

*D*—H···*A*	*D*—H	H···*A*	*D*···*A*	*D*—H···*A*
N1—H1*N*···O1	0.87 (2)	2.217 (19)	2.9568 (15)	142.2 (16)
N1—H1*N*···Cl2	0.87 (2)	2.536 (19)	2.9890 (13)	113.1 (15)
C15—H15*A*···O2^i^	0.95 (2)	2.59 (2)	3.521 (2)	168.2 (15)
C2—H2···N1^ii^	0.96 (2)	2.93 (2)	3.886 (2)	173.6 (16)

Symmetry codes: (i) −*x*+1, *y*−1/2, −*z*+3/2; (ii) *x*, −*y*+1/2, *z*−1/2.
