# Supplementary material for: Crystal structure of chloro­methyl 2-[2-(2,6-di­chloro­phenyl­amino)­phen­yl]acetate
Source: Acta Crystallogr E Crystallogr Commun. 2025 May 13;81(Pt 6):510–5. doi: 10.1107/S2056989025004074 (PMC12142405; doi:10.1107/S2056989025004074)
Supplement: Supplementary file 3 [file e-81-00510-sup3.pdf]

**Supporting Information**

# Crystal structure of chloromethyl 2-[2-(2,6-di-chloroanilino)phenyl]acetate

Tobias Keydel,[a] Siva S. M. Bandaru,[b] Lukas Schulig,[a] Andreas Link[a]* and Carola Schulzke[b]*

[a]Institute of Pharmacy, University of Greifswald, Friedrich-Ludwig-Jahn-Straße 17, 17489 Greifswald, Germany, and
[b]Institute of Biochemistry, University of Greifswald, Felix-Hausdorff-Straße 4, 17489 Greifswald, Germany
Correspondence email: carola.schulzke@uni-greifswald.de

# 1 General Experimental Information

The starting materials, reagents, and solvents were commercially available and purchased from TCI or ABCR. All chemicals were used as received unless specified otherwise. NMR spectra were recorded on a Bruker Avance III device at 400 MHz ($^1$H) and 100 MHz ($^{13}$C), respectively, using DMSO-$d_6$ as solvent. The chemical shifts were referenced to the internal standard tetramethylsilane (TMS) and reported in parts per million (ppm). The coupling constants ($J$) are in Hz, and the following abbreviations were used to designate the multiplicities: s (singlet), d (doublet), t (triplet), dd (doublet of doublet), m (multiplet). A Bruker Elute UHPLC with Bruker compact QTOF-MS operated with ESI ionization, were used to measure the HRAM-MS data. The melting point was measured with an automated Büchi Melting Point M-565 device. Analytical thin-layer chromatography was carried out on silica gel 60 $F_{254}$ aluminum plates obtained from Merck, and visualization was accomplished with UV light. Dry column vacuum chromatography on silica gel was performed using silica gel 60 from Carl Roth with a particle size of 20–45 µm.

# 2 NMR Data

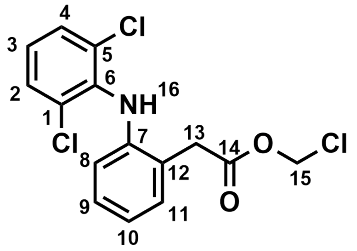


**$^1$H NMR** (400 MHz, DMSO-$d_6$): δ 7.53 (d, $J$ = 8.1 Hz, 2H (**2**, **4**)), 7.28 – 7.17 (m, 2H (**9**, **11**)), 7.07 (td, $J$ = 7.7, 1.6 Hz, 1H (**3**)), 7.01 (s, 1H, (**16**)), 6.84 (td, $J$ = 7.4, 1.2 Hz, 1H (**10**)), 6.23 (ddd, $J$ = 8.2, 3.3, 1.3 Hz, 1H (**8**)), 5.89 (s, 2H (**15**)), 3.94 (s, 2H (**13**));
**$^{13}$C NMR** (101 MHz, DMSO-$d_6$): δ 169.70 (**14**), 142.98 (**7**), 137.00 (**6**), 131.28 (**1**, **5**), 131.05 (**11**), 129.12 (**2**, **4**), 127.99 (**3**), 126.23 (**9**), 121.88 (**12**), 120.41 (**10**), 115.60 (**8**), 69.74 (**15**), 36.33 (**13**);

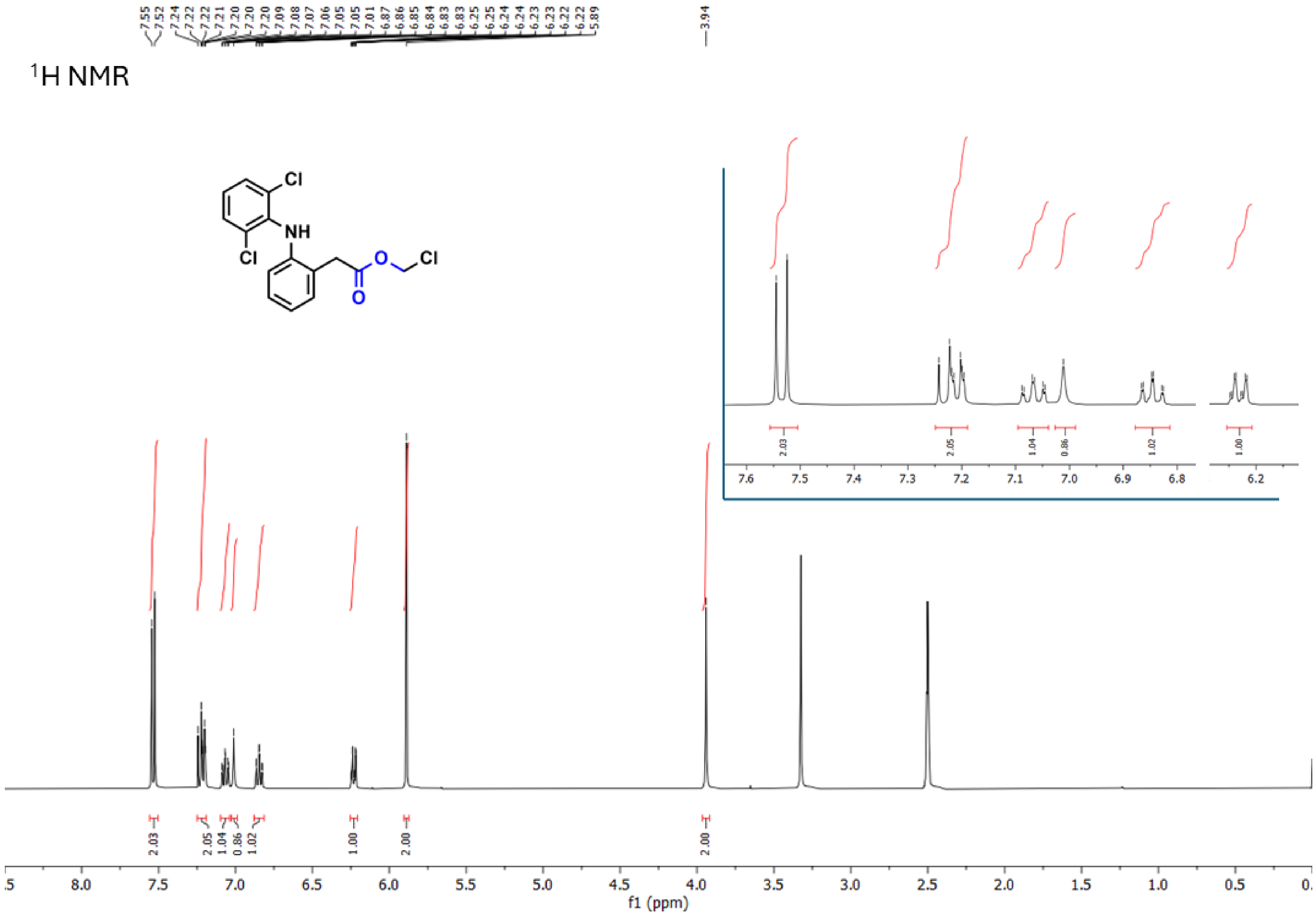

7.55
7.52
7.24
7.22
7.22
7.21
7.20
7.20
7.20
7.09
7.08
7.07
7.06
7.05
7.05
7.01
6.87
6.86
6.85
6.84
6.83
6.83
6.25
6.25
6.24
6.24
6.23
6.23
6.22
6.22
5.89
3.94
1H NMR
Cl
NH
Cl
O
Cl
O
2.03
2.05
1.04
0.86
1.02
1.00
2.00
2.00
f1 (ppm)

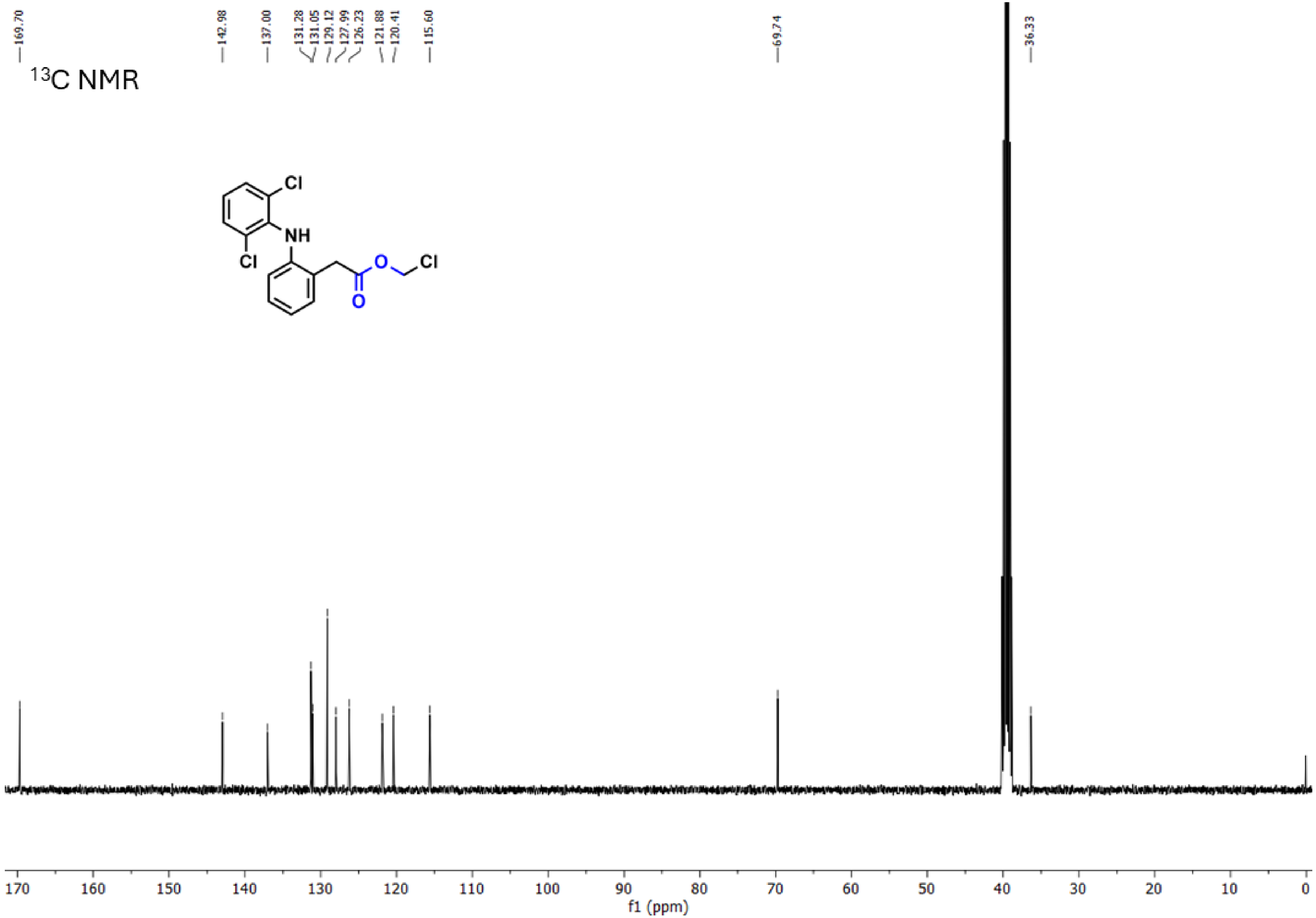
169.70
142.98
137.00
131.28
131.05
129.12
127.99
126.23
121.88
120.41
115.60
69.74
36.33
$^{13}C$ NMR
Cl
NH
Cl
O
Cl
O
170 160 150 140 130 120 110 100 90 80 70 60 50 40 30 20 10 0
f1 (ppm)
